# Genistein suppresses leptin‐induced proliferation and migration of vascular smooth muscle cells and neointima formation

**DOI:** 10.1111/jcmm.12986

**Published:** 2016-09-28

**Authors:** Yung‐Chieh Tsai, Sy‐Ying Leu, Yi‐Jen Peng, Yen‐Mei Lee, Chih‐Hsiung Hsu, Shen‐Chieh Chou, Mao‐Hsiung Yen, Pao‐Yun Cheng

**Affiliations:** ^1^ Department of Obstetrics and GynecologyChi‐Mei Medical CenterTainanTaiwan; ^2^ Department of MedicineTaipei Medical UniversityTaipeiTaiwan; ^3^ Department of Sport ManagementChia Nan University of Pharmacy and ScienceTainanTaiwan; ^4^ Graduate Institute of Life SciencesNational Defense Medical CenterTaipeiTaiwan; ^5^ Department of PathologyTri‐Service General Hospital and National Defense Medical CenterTaipeiTaiwan; ^6^ Department of PharmacologyNational Defense Medical CenterTaipeiTaiwan; ^7^ Department of Internal MedicineTri‐Service General HospitalNational Defense Medical CenterTaipeiTaiwan; ^8^ Department of Biological Science and TechnologyCollege of Biopharmaceutical and Food SciencesChina Medical UniversityTaichungTaiwan; ^9^ Department of Physiology and BiophysicsGraduate Institute of PhysiologyNational Defense Medical CenterTaipeiTaiwan

**Keywords:** genistein, Leptin, MMP2, carotid artery injury, reactive oxygen species

## Abstract

Obesity is a strong risk factor for the development of cardiovascular diseases and is associated with a marked increase in circulating leptin concentration. Leptin is a peptide hormone mainly produced by adipose tissue and is regulated by energy level, hormones and various inflammatory mediators. Genistein is an isoflavone that exhibits diverse health‐promoting effects. Here, we investigated whether genistein suppressed the atherogenic effect induced by leptin. The A10 cells were treated with leptin and/or genistein, and then the cell proliferation and migration were analysed. The reactive oxygen species (ROS) and proteins levels were also measured, such as p44/42MAPK, cell cycle‐related protein (cyclin D1 and p21) and matrix metalloproteinase‐2 (MMP‐2). Immunohistochemistry and morphometric analysis were used for the neointima formation in a rat carotid artery injury model. Genistein (5 μM) significantly inhibited both the proliferation and migration of leptin (10 ng/ml)‐stimulated A10 cells. In accordance with these finding, genistein decreased the leptin‐stimulated ROS production and phosphorylation of the p44/42MAPK signal transduction pathway. Meanwhile, genistein reversed the leptin‐induced expression of cyclin D1, and cyclin‐dependent kinase inhibitor, p21. Genistein attenuated leptin‐induced A10 cell migration by inhibiting MMP‐2 activity. Furthermore, the leptin (0.25 mg/kg)‐augmented neointima formation in a rat carotid artery injury model was attenuated in the genistein (5 mg/kg body weight)‐treated group when compared with the balloon injury plus leptin group. Genistein was capable of suppressing the atherogenic effects of leptin *in vitro* and *in vivo*, and may be a promising candidate drug in the clinical setting.

## Introduction

The proliferation and migration of vascular smooth muscle cells (VSMCs) plays a vital role in arterial intimal thickening, vascular remodelling, atherosclerosis and restenosis after balloon angioplasty and hypertension 1. There are many risk factors that could enhance the process, such as obesity 2, 3. Leptin is a product of the obesity gene and is mainly secreted by adipocytes and is involved in the regulation of appetite and energy metabolism 4, 5. It has been demonstrated positively to be associated with clinical cardiovascular disease such as hypertension, myocardial infarction and neointimal hyperplasia 6, 7, 8. Leptin receptors are expressed in atherosclerotic plaque, injured neointima and the media 9 and are found in VSMCs. Leptin binds to its receptor and promotes atherogenesis processes, including platelet aggregation, inflammation, endothelial dysfunction and VSMC proliferation and migration 8. Therefore, the inhibition of leptin‐induced VSMC proliferation and migration may represent a therapeutic intervention in atherosclerosis after obesity.

Genistein is a phytoestrogen, a plant‐derived estrogenic compound, and belongs to the group of isoflavones 10. Several studies have shown that genistein exerts several beneficial effects such as prevention of breast and cardiovascular diseases and attenuation of osteoporosis and other post‐menopausal symptoms 11, 12, 13, 14. Its action involves inducing nitric oxide synthesis 11, 12, improving the resistance of low‐density lipoprotein against *ex vivo* oxidation 13 and improving flow‐mediated endothelium‐dependent dilatation and increases in the ratio of nitric oxide to endothelium, which is a parameter for endothelial function 14. Its anti‐oxidant property has been reported 15. However, the inhibitory effect of genistein on VSMC neointima has not been studied extensively. Therefore, the aim of this study was to investigate the effects of genistein on the proliferation and migration of VSMCs induced by leptin and on neointima formation of the carotid artery.

## Materials and methods

### Cell culture

Rat aortic smooth muscle A10 cells (derived from the American Type Culture Collection) were purchased from the Food Industry Research and Development Institute, Hsinchu, Taiwan. The cells were cultured in DMEM (Gibco Life Technologies, Grand Island, NY, USA) supplemented with 10% foetal bovine serum (Gibco Life Technologies) at 37°C in a humidified atmosphere of 5% CO_2_. Cells were used between passages 6 and 18 for all experiments.

### Cell proliferation assay

Cells were treated with leptin (1–100 ng/ml; R&D Systems, Inc., Minneapolis, MN, USA) for 72 hrs and/or pre‐treated with genistein (1, 5 μM; Sigma‐Aldrich, St Louis, MO, USA) for 1 hr, and the relative cell numbers were assessed using the MTS‐based CellTiter 96^®^ AQueous One Solution kit (Promega, Madison, WI, USA), according to the manufacturer's directions 16.

### Bromodeoxyuridine incorporation assay

The proliferation of cells was also measured by DNA synthesis using a bromodeoxyuridine (BrdU) proliferation assay kit (Cell Signaling Technology, Danvers, MA, USA), according to the manufacturer's directions, as described previously 16.

### Lactate dehydrogenase release assay

The cells were treated with different concentration of genistein for 24 hrs, and then the medium was collected to measure the amount of released lactate dehydrogenase (LDH) by an LDH cytotoxicity assay kit (BioChain, Thurmont, MD, USA), according to the manufacturer's directions.

### Cell migration assay

Cell migration assay was performed with the Transwell^®^ Permeable Support Culture Plate System (Corning Inc., Corning, NY, USA) as described previously 16.

### Western blot analysis

Samples were run out in 10% SDS‐PAGE, subsequently transferred to nitrocellulose membrane (Millipore, Bedford, MA, USA) and blocked in Tris‐buffered saline (10 mmol/l Tris‐HCl, 150 mmol/l NaCl, pH 8.00) with 0.05% Tween 20 (TBS‐T) containing 5% non‐fat dry milk for 1 hr at room temperature. Blots were then incubated overnight at 4°C with rabbit anti‐phosphorylated p44/42MAPK (1:1000 dilution; Cell Signaling Technology), rabbit anti‐cyclin D1 (1:2000 dilution; Santa Cruz Biotechnology, Dallas, TX, USA), mouse anti‐p21 (1:2000 dilution; Santa Cruz Biotechnology), anti‐MMP‐2 (1:1000 dilution; Millipore, Temecula, CA, USA) and mouse anti‐β‐actin (1:2000 dilution; Sigma‐Aldrich) antibodies. The membranes were incubated with HRP‐conjugated secondary antibodies (1:1000 dilution; Cell Signaling Technology). The blots were detected with an enhanced chemiluminescence kit (Pierce, Rockford, IL, USA) and a bio‐imaging analyser (Fujifilm LAS‐4000; GE Healthcare Life Sciences, Marlborough, MA, USA). Densitometric analysis was conducted with Image‐Pro software (Media Cybermetrics, Inc., Bethesda, MD, USA).

### Gelatin zymography

For the measurement of MMP‐2 activity of culture media, gelatin zymography was conducted. Briefly, culture media were subject to electrophoresis on a Novex 10% gelatin zymogram gel (Invitrogen, Life Technologies, Carlsbad, CA, USA) as described previously 16.

### Detection of intracellular reactive oxygen species

The intracellular reactive oxygen species (ROS) was determined using a CellROX Green Reagent (Life Technologies), according to the manufacturer's directions. Cells treated with leptin for 1 hr and/or pre‐treated with genistein for 1 hr were incubated in DMEM containing 5 μM CellROX Green Reagent for 1 hr at 37°C in the dark. Cells were then fixed with 3.7% formaldehyde in PBS at RT for 15 min. Cells were washed in PBS three times between each step. The green fluorescence signals were detected with a laser scanning confocal microscope (Zeiss LSM 510; Carl Zeiss, Jena, Germany) and an inverted microscope (Axiovert 100; Carl Zeiss) with a 60 × 1.4 numerical aperture oil immersion objective as described previously 17.

### Animals

Male Sprague–Dawley rats, weighing 250–300 g, were used for the study. This study was approved by the Institutional Animal Care and Use Committee of China Medical University, Taiwan. All animals were obtained from the National Laboratory Animal Breeding and Research Center of the National Science Council, Taiwan, and were handled in accordance with the guide for the Care and Use of Laboratory Animals (National Academic Press, Washington, DC, 1996). Animals were fed standard rodent chow and water.

### Rat carotid balloon injury and leptin and genistein administration

The rat carotid artery model of balloon angioplasty was established to examine the *in vivo* arterial response to injury 18. Briefly, male Sprague–Dawley rats were divided into four groups: (*i*) Sham (*n* = 2); (*ii*) Balloon injury (*n* = 2); (*iii*) Balloon injury plus leptin (0.25 mg/kg body weight in 100 μl normal saline, twice daily, i.p.; R&D system) (*n* = 3) and (*iv*) Balloon injury plus leptin and genistein (5 mg/kg body weight, i.p.; Sigma‐Aldrich) (*n* = 3). The doses of leptin and genistein were chosen according to Schäfer *et al*. 19 and Menze *et al*. 20, respectively. After intraperitoneal anaesthesia with Zoletil (20 mg/kg; Virbac Co, Carros, France), the left carotid artery was exposed. A Fogarty 2F embolectomy balloon catheter (Edwards Lifesciences, Irvine, CA, USA) was inserted into the left external carotid artery *via* arteriotomy, and the balloon was inflated with saline and drawn towards the arteriotomy site five times to produce a distending and de‐endothelializing injury 21. Fourteen days after balloon injury, the rats were killed, and sections from left carotid arteries were excised and fixed with 10% formalin for morphometric and immunohistochemical analysis.

### Histological and morphometric analysis

For immunohistochemistry and morphometric analysis, the arteries were fixed in 10% formalin for 24 hrs, and then the middle one third of the common carotid artery was cut into four segments and embedded in paraffin. The specimens were cross‐sectioned at a thickness of 4 μm and stained with haematoxylin and eosin. Smooth muscle cells were detected by smooth muscle α‐actin staining using an α‐actin monoclonal antibody (1:100; Cedarlane Laboratories Ltd, Hornby, Ontario, Canada).

### Statistical analysis

All measurements are expressed as means ± S.E.M. Statistical evaluation was performed with one‐way anova followed by the Newman–Keuls method. A *P*‐value of less than 0.05 was deemed statistically significant.

## Results

### Inhibitory effects of genistein on leptin‐induced proliferation of A10 cells

**Figure 1:**
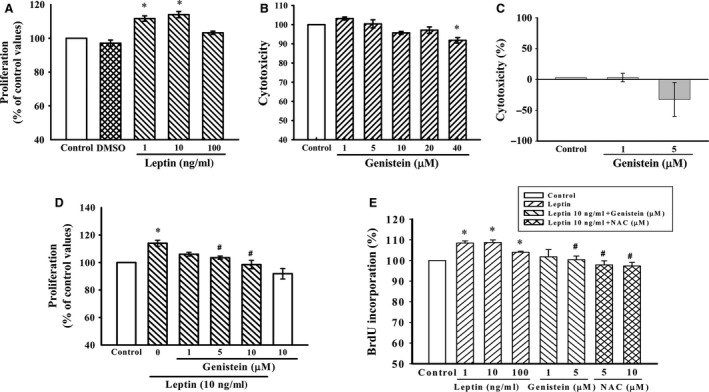
Effect of genistein on leptin‐induced proliferation of A10 cells. (**A** and **D**) Growth‐arrested cells were stimulated with leptin (1–100 ng/ml) for 72 hrs in the presence or absence of genistein (1–10 μM). Cell proliferation was assayed by the CellTiter 96^®^ AQueous One Solution kit. Relative proliferation activities were determined using untreated control cells as a standard. Data represent the mean ± S.E.M. of six independent observations with different cell passages and on different days. *P < 0.05 *versus* Control; ^#^ *P* < 0.05 *versus* Leptin (10 ng/ml) alone. (**B** and **C**) Cells were incubated with genistein at increasing concentrations (1–40 μM) for 24 hrs; the toxic effects of genistein were measured by the CellTiter 96^®^ AQueous One Solution kit and LDH cytotoxicity assay kit, respectively. Data represent the mean ± S.E.M. of four independent observations with different cell passages and on different days. *P < 0.05 *versus* Control. (**E**) DNA synthesis was measured by the BrdU incorporation assay. Growth‐arrested cells were stimulated with leptin (1–100 ng/ml) for 72 hrs in the presence or absence of genistein (1 and 5 μM) or NAC (5 and 10 μM). Data represent the mean ± S.E.M. of six independent observations with different cell passages and on different days. *P < 0.05 *versus* Control; ^#^ *P* < 0.05 *versus* Leptin (10 ng/ml) alone.

Exposure of A10 cells to various concentration leptin (1, 10 and 100 ng/ml) for 72 hrs resulted in a significant increase in the number of cells, with maximal levels at 10 ng/ml (Fig. 1A). Therefore, the 10 ng/ml concentration of leptin was selected for further studies. Compared to the control group, genistein (1, 5, 10 and 20 μM) had no effect on cell proliferation. However, A10 cell proliferation was significantly inhibited by genistein (40 μM) and was 10% lower than that observed for the controls (Fig. 1B). The concentrations of genistein used did not exhibit cytotoxic effects on cell viability; therefore, the 1–10 μM concentration range of genistein was used in the following studies. The LDH cytotoxicity analysis was also used for confirming the absence of cytotoxicity of genistein (Fig. 1C). We next studied the effect of genistein on the proliferation of A10 cells induced by leptin using the MTS Assay and the DNA synthesis by BrdU incorporation assay. The proliferation of A10 cells stimulated with leptin (10 ng/ml) was significantly attenuated when pre‐treated with genistein (5, 10 μM) (Fig. 1D and E). The effect of leptin on the cell proliferation was also inhibited when pre‐treated with N‐acetylcysteine (NAC, 5 and 10 μM), a free radical scavenger, was examined by using the BrdU incorporation assay (Fig. 1E).

### Effect of genistein on p44/42MAPK phosphorylation in leptin‐stimulated A10 cells

**Figure 2:**
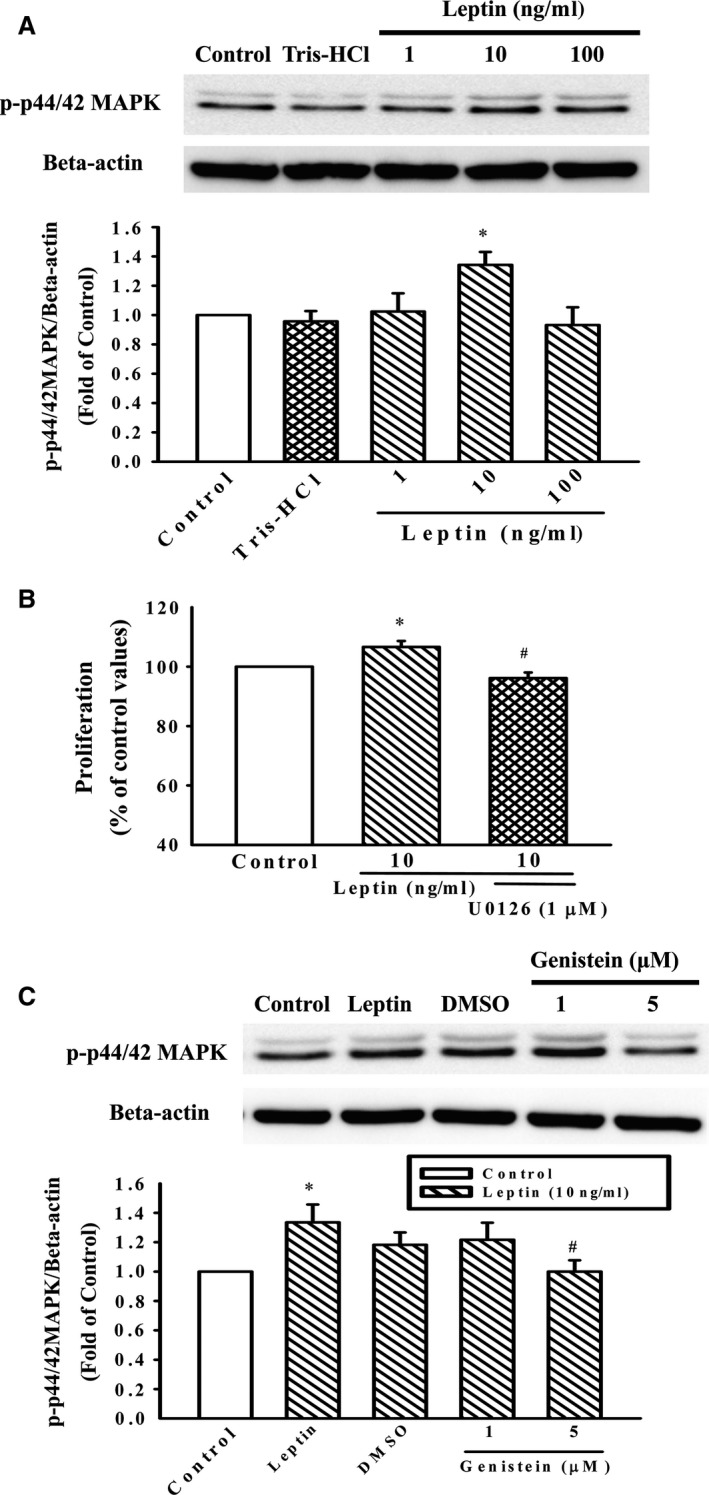
Effect of genistein on leptin‐induced p44/42MAPK phosphorylation in A10 cells. (**A** and **C**) Cells were stimulated with leptin (1–100 ng/ml) for 30 min. in the presence or absence of genistein (1 and 5 μM). The cells were lysed and proteins were analysed by Western blotting. β‐actin was used for normalization. Data represent the mean ± S.E.M. of five independent observations with different cell passages and on different days. *P < 0.05 *versus* Control; ^#^ *P* < 0.05 *versus* Leptin (10 ng/ml) alone. (**B**) Growth‐arrested cells were stimulated with leptin (10 ng/ml) for 72 hrs in the presence or absence of U0‐126 (1 μM). Cell proliferation was assayed by the CellTiter 96^®^ AQueous One Solution kit. Relative proliferation activities were determined using untreated control cells as a standard. Data represent the mean ± S.E.M. of six independent observations with different cell passages and on different days. *P < 0.05 *versus* Control; ^#^ *P* < 0.05 *versus* Leptin (10 ng/ml) alone.

Leptin significantly induced the phosphorylation of p44/42MAPK in A10 cells and the phosphorylated p44/42MAPK reached maximum levels when the cells were treated with 10 ng/ml leptin (Fig. 2A). To address the role of p44/42MAPK in VSMCs proliferation stimulated by leptin, we evaluated the effect of UO‐126 (MEK‐1 inhibitor) on the induction of cell proliferation by leptin. Leptin‐stimulated A10 cell proliferation was significantly attenuated by pre‐treatment with U0126 (1 μM) for 1 hr (Fig. 2B). Next, the phosphorylation of p44/42MAPK induced by leptin was significantly inhibited by the pre‐treatment of genistein (5 μM; Fig. 2C).

### Effect of genistein on cyclin D1 and p21 expression in A10 cells

**Figure 3:**
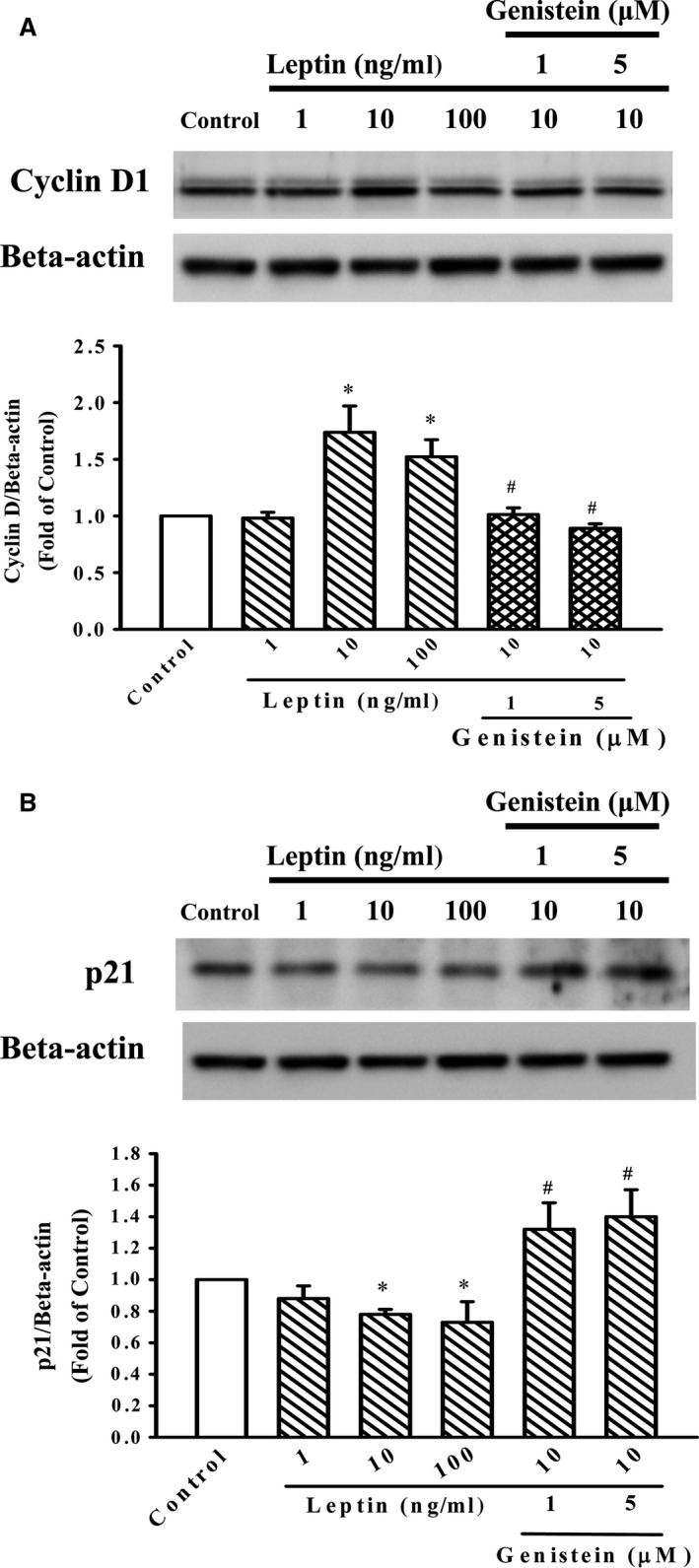
Effect of genistein on leptin‐induced cyclin D1 and p21 protein expression in A10 cells. (**A** and **B**) Cells were stimulated with leptin (1–100 ng/ml) for 3 hrs in the presence or absence of genistein (1 and 5 μM). The cells were lysed and both cyclin D1 and p21 proteins were analysed by Western blotting. β‐actin was used for normalization. Data represent the mean ± S.E.M. of three independent observations with different cell passages and on different days. *P < 0.05 *versus* Control; ^#^ *P* < 0.05 *versus* Leptin (10 ng/ml) alone.

To clarify whether the cell cycle progression involved in the anti‐proliferative effect of genistein, the cell cycle‐related protein was analysed. Leptin (10–100 ng/ml) significantly induced the expression of cyclin D1 in A10 cells and the cyclin D1 reached maximum levels when the cells were stimulated with 10 ng/ml leptin (Fig. 3A). In addition, the p21 protein level was significantly attenuated by leptin (10–100 ng/ml) (Fig. 3B). However, these patterns of leptin were all reversed by genistein (1 and 5 μM) treatment.

### Inhibitory effects of genistein on leptin‐induced cell migration

**Figure 4:**
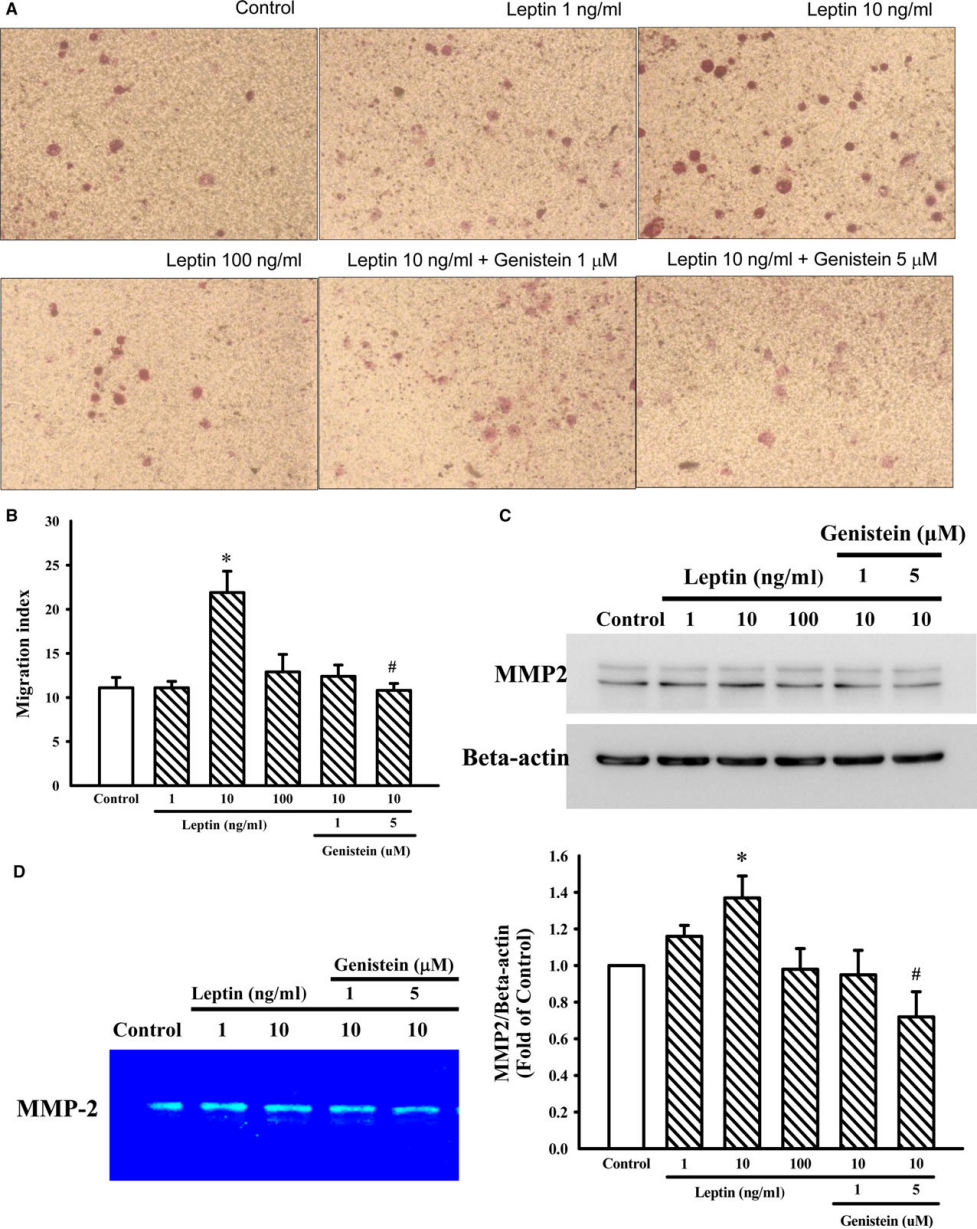
Effects of genistein on leptin‐induced VSMC migration as well as MMP‐2 protein expression in A10 cells. (**A** and **B**) VSMC migration was examined using the Transwell^®^ Permeable Support Culture Plate System. Genistein (1 and 5 μM) was added to both the upper and the lower compartments and was present throughout the duration of the experiment. Migration was induced by the addition of leptin (10 ng/ml) to the lower chamber. After incubation at 37°C for 48 hrs, the non‐migratory cells were removed from the upper surface of the membrane by scraping them with cotton swabs. The membrane was fixed with 90% ethanol and stained with 0.1% crystal. Migrated cells were counted at 200× magnification in five randomly chosen microscope fields per filter. The figure (**B**) indicates the fold value of cell migration. Data represent the mean ± S.E.M. of four independent observations with different cell passages and on different days. (**C**) Cells were stimulated with leptin (1–100 ng/ml) for 3 hrs in the presence or absence of genistein (1 and 5 μM). The cells were lysed and MMP‐2 protein was analysed by Western blotting. β‐actin was used for normalization. Data represent the mean ± S.E.M. of five independent observations with different cell passages and on different days. *P < 0.05 *versus* Control; ^#^ *P* < 0.05 *versus* Leptin (10 ng/ml) alone. (**D**) Gelatin zymography analysis was performed with conditioned media collected from A10 cells cultured in the presence or absence of genistein (1 and 5 μM) and leptin (10 ng/ml).

The cell migration activity was significantly increased by the stimulation of leptin (10–100 ng/ml) in A10 cells, with a maximal effect at 10 ng/ml (Fig. 4A and B). However, the migration effect of leptin was significantly attenuated by pre‐treatment with genistein (5 μM).

### Effect of genistein on MMP‐2 protein expression in A10 cells

Matrix metalloproteinase‐2 had been implicated in VSMC migration to the intima *via* the degradation of extracellular matrix. The effect of genistein on the expression of MMP2 was further investigated. Genistein (5 μM) significantly attenuated the up‐regulation of MMP‐2 induced by leptin (10 ng/ml) in A10 cells (Fig. 4C). Meanwhile, the MMP‐2 proteolytic activity in leptin culture medium was also attenuated when the cells were pre‐treated with genistein (Fig. 4D).

### Effects of genistein alone on protein expression in A10 cells

**Figure 5:**
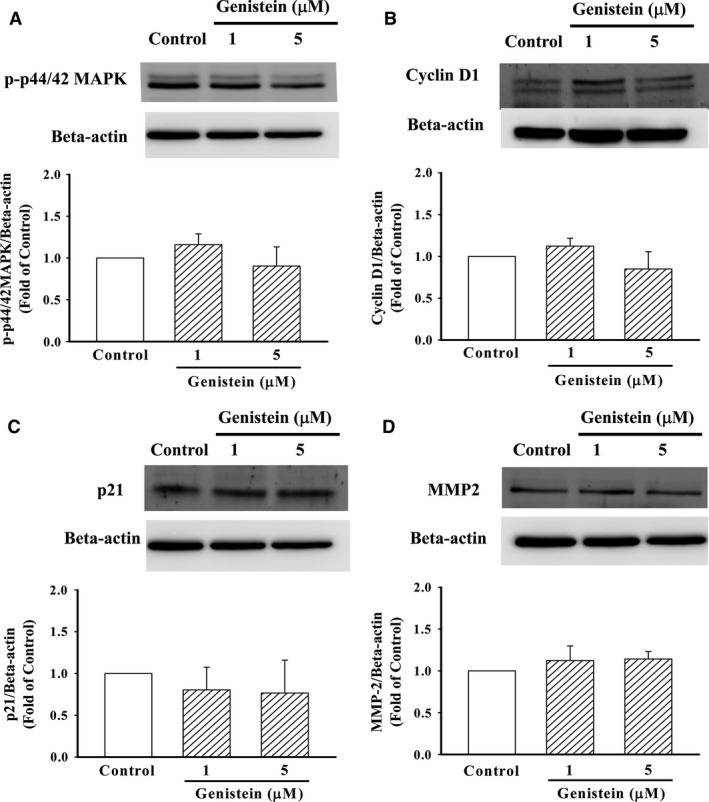
Effects of genistein alone on phosphorylated p44/42MAPK, cyclin D1, p21 and MMP2 protein expression in A10 cells. (**A**–**D**) Cells were stimulated with genistein (1, 5 μM) for 24 hrs. The cells were lysed and proteins were analysed by Western blotting. β‐actin was used for normalization. Data represent the mean ± S.E.M. of three independent observations with different cell passages and on different days. *P < 0.05 *versus* Control.

To further clarify the above effects of genistein on the pattern in cells raised by leptin, the effect of genistein alone on phosphorylation and proteins expression in A10 cells were evaluated. As shown in Figure 5, genistein alone had a neutral effect on phosphorylation of p44/42MAPK, as well as cyclin D1, p21 and MMP2 protein expression similar to control cells.

### Effect of genistein on leptin‐induced production of ROS

**Figure 6:**
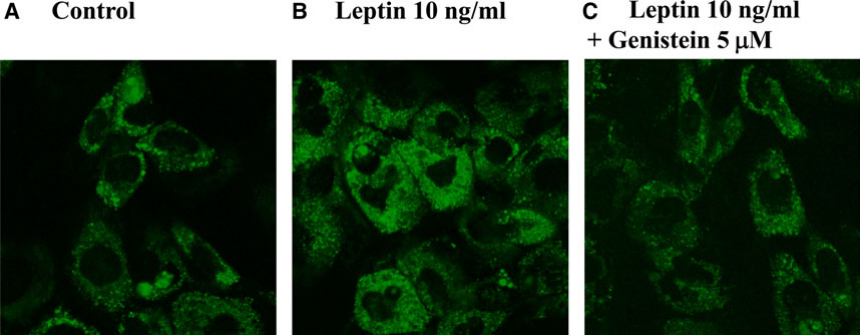
Effects of genistein on leptin‐induced ROS production in A10 cells. Cells were stimulated with leptin (10 ng/ml) for 1 hr in the presence or absence of genistein (5 μM). Representative images showing that intracellular ROS production was detected using CellROX Green Reagent.

To evaluate the effect of genistein on leptin‐induced production of ROS, the CellROX Green Reagent was used for the measurement of ROS. As shown in Figure 6, exposure to leptin induced the production of ROS, as expected (Fig. 6B), and pre‐incubation with genistein suppressed ROS production induced by leptin (Fig. 6C).

### Effect of genistein on leptin‐enhanced neointimal hyperplasia after balloon injury

**Figure 7:**
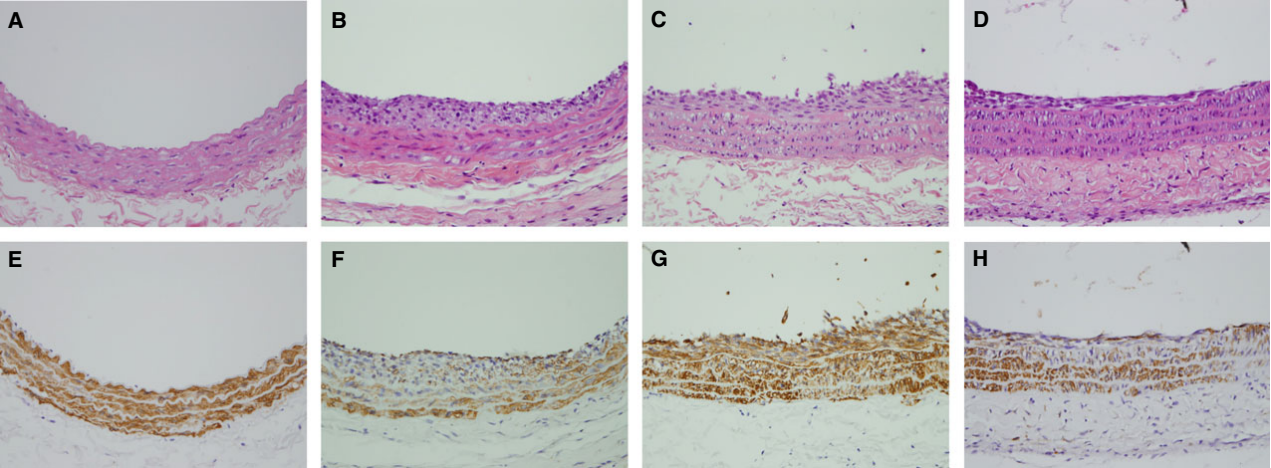
Typical examples of carotid arteries of rats treated with leptin alone or co‐treated with genistein after balloon injury. (**A** and **E**) Sham surgery, (**B** and **F**) arterial ballooning injury, (**C** and **G**) arterial ballooning injury then leptin (0.25 mg/kg body weight, i.p.) treatment, (**D** and **H**) arterial ballooning injury then leptin and genistein (5 mg/kg body weight, i.p.) treatment, showing significant attenuation of neointimal smooth muscle cell proliferation. **A**–**D** (haematoxylin and eosin stain 400×) compared with **E**–**H** (immuohistochemical stain of anti‐alpha smooth muscle actin, 400×).

Neointimal smooth muscle proliferation in injured vessels was observed morphologically (Fig. 7B; haematoxylin and eosin, 400×) and highlighted by an immunohistochemical staining of alpha‐smooth muscle actin (Fig. 7F; 400×). The injured vessels treated with leptin (0.25 mg/kg body weight) showed marked neointimal smooth muscle proliferation (Fig. 7C and G; 400×). Genistein (5 mg/kg body weight) significantly inhibited the accumulation of neointimal smooth muscle cells in injured vessels treated with leptin at 14 days (Fig. 7D and H; 400×).

## Discussion

In this study, we demonstrated, for the first time, that genistein has an anti‐atherogenic effect on aortic smooth muscle cells stimulated by leptin both *in vitro* and *in vivo*. Our data also indicated that genistein may function by the up‐regulation of p21 expression, suppression of ROS production and down‐regulation of phosphorylated p44/42MAPK, cyclin D1 and MMP‐2 expression and that inhibiting leptin‐enhanced neointimal formation may be a potential therapeutic strategy for the prevention, possibly, treatment of vascular diseases.

Clinical and experimental evidence suggests that the adipokine leptin may directly link obesity with the elevated cardiovascular risk associated with increased body weight 9, 19, 22. For example, it had been found that db/db mice (which lack the functional leptin receptor) did not develop neointimal hyperplasia despite the presence of obesity 22, whereas exogenous administration of leptin promoted experimental lesion formation in injured arteries from wild‐type mice but had no effect on vessels from leptin receptor‐deficient db/db mice 19. In this study, we found that leptin could induce the proliferation and migration of A10 cells in a dose‐dependent manner (Figs 1A and 4A, B). Meanwhile, genistein potently reduced this pattern in cells raised by leptin (Figs 1C–E and 4A, B), and the anti‐atherogenic effect of genistein was not due to its cytotoxicity (Fig. 1B). In addition, leptin increased neointimal hyperplasia and this was confirmed in a rat carotid arterial injury model (Fig. 7C and G). Genistein treatment attenuated leptin‐enhanced neointimal hyperplasia of the carotid artery after balloon injury (Fig. 7D and H). These results suggest that genistein maybe a good candidate for the prevention of neointimal hyperplasia and atherosclerosis in obesity.

The p44/42MAPK are serine/threonine kinases activated by a variety of stimuli involved in cell proliferation and differentiation 23, 24. Research groups have reported that p44/42MAPK is activated in the arterial wall following balloon injury in a number of animal models 23, 24, 25. These findings suggest that p44/42MAPK could represent a link between arterial injury and VSMC proliferation in atherosclerotic diseases and, therefore, leptin‐induced p44/42MAPK activation could be involved in the pathogenesis of atherosclerosis. In this study, the leptin‐activated p44/42MAPK was attenuated by the pre‐treatment of genistein, and the role of p44/42MAPK in the proliferation was further confirmed by the MEK1/2 inhibitor (Fig. 2B). Therefore, the inhibition of p44/42MAPK may contribute to the anti‐atherogenic effect of genistein.

Generally, the cell cycle is regulated by the coordinated action of cyclin‐dependent kinases (CDK) in association with their specific regulatory cyclin proteins. It was reported that synthesis of cyclin D1 may be the target of physiological signals that control cell proliferation 26. In addition, p21 is a universal inhibitor of cyclin/CDK catalytic activity and arrests cell growth. This study had showed that genistein significantly reversed the effect of leptin on the expression of cyclin D1 and p21 (Fig. 3). The expression of p21 up‐regulated by genistein is consistent with its inhibitory effect on cyclin D1. Thus, blocking of cell cycle progression may contribute to the anti‐proliferative effect of genistein.

Early studies showed that MMPs play an important role in atherosclerotic lesion progression. In addition, migration of VSMCs may require the degradation or remodelling of extracellular matrix surrounding the cells 27. This study revealed that genistein treatment reduced leptin‐enhanced neointimal hyperplasia of the carotid artery (Fig. 7C, D, G and H). *In vitro* study, we also found that genistein reduced migration of leptin‐stimulated cells, which is concomitant with reduced MMP‐2 activity (Fig. 4). Therefore, genistein inhibited leptin‐stimulated cell migration by attenuating MMP‐2 expression and enzymatic activity, which have been linked to the intimal formation in arterial lesions.

Under various pathological conditions, ROS and MAPK may contribute to the vascular remodelling 28. Previous study had also report that leptin induced the production of intracellular ROS in VSMCs and ROS generation was related to activation of MAPKs, which promote cellular proliferation and migration 29, 30. These results supported the possibility that ROS‐p44/42MAPK pathway may play an important role in leptin‐induced smooth muscle cell proliferation. This study revealed that NAC (ROS scavenger) mimicked the inhibitory effects of genistein on the leptin‐stimulated cell proliferation (Fig. 1E), and the leptin‐induced production of ROS was attenuated by pre‐treatment with genistein (Fig. 6C). These results support previous report that genistein has antioxidant properties 15. Together with our observation, it is possible that the antioxidant effect of genistein may involve in the anti‐proliferatory effect of leptin.

In this study, the effect of leptin on the cell proliferation, migration and related proteins expression (p44/42 MAPK, cyclin D and MMP‐2) was investigated in A10 cells. Leptin alone had a biphasic effect on these targets (Figs 1A, 2A, 3A and 4B). A relatively low concentration of 10 ng/ml leptin significantly increased the cell proliferation, migration and proteins expression, whereas 100 ng/ml leptin produces less effect. Although we have no direct evidence of the differences in leptin on these phenomenon, we speculate that (*i*) leptin can increase the cell proliferation, migration and related proteins expression indirectly, possibly by activating or inhibiting another factor, to decrease these patterns and (*ii*) leptin may acts as an inhibitory regulator of its own receptor isoforms in VSMCs 31. Further studies are needed to clarify the mechanism of different concentration of leptin on the cell proliferation, migration and related proteins expression.

## Conclusion

Genistein effectively inhibits leptin‐induced proliferation and migration in cultured A10 cells. In addition, the effects exerted by genistein may associate with the inhibition of p44/42MAPK, cyclin D1 and MMP‐2 expression, attenuation of ROS production and the induction of p21. These effects were also applicable *in vivo* for inhibiting neointimal formation after ballooning arterial injury. These findings provide insights into the potential novel effect of genistein as a therapeutic agent against vascular diseases after obesity.

## Conflicts of interest

The authors declare that they have no competing financial interests.

## Author contribution

S.Y.L., Y.J.P. and Y.C.T. acquired data; Y.C.T., C.H.H., M.H.Y. and P.Y.C. contributed to the initial discussion of the project; Y.M.L., S.C.C. and P.Y.C. reviewed the article; Y.C.T. and P.Y.C. wrote the article.
